# TILRR (FREM1 isoform 2) is a prognostic biomarker correlated with immune infiltration in breast cancer

**DOI:** 10.18632/aging.103798

**Published:** 2020-10-08

**Authors:** Xiao-Yi Xu, Wen-Jing Guo, Shi-Hua Pan, Ying Zhang, Feng-Lin Gao, Jiang-Tao Wang, Sheng Zhang, He-Ying Li, Ren Wang, Xiao Zhang

**Affiliations:** 1 CAS Key Laboratory of Regenerative Biology, Joint School of Life Sciences, Guangzhou Institutes of Biomedicine and Health, Chinese Academy of Sciences, and Guangzhou Medical University, Guangzhou, Guangdong, China; 2 Guangzhou Regenerative Medicine and Health Guangdong Laboratory, Guangzhou 510530, Guangdong, China; 3 Guangdong Provincial Key Laboratory of Biocomputing, Guangzhou Institutes of Biomedicine and Health, Chinese Academy of Sciences, Guangzhou 510530, Guangdong, China; 4 Guangdong Provincial Key Laboratory of Stem Cell and Regenerative Medicine, South China Institute for Stem Cell Biology and Regenerative Medicine, Guangzhou Institutes of Biomedicine and Health, Chinese Academy of Sciences, Guangzhou 510530, Guangdong, Guangdong, China; 5 Affiliated Cancer Hospital and Institute, Guangzhou Medical University, Guangzhou 511436, Guangdong, China; 6 Department of Pathology, First People Hospital, Changde 415003, Hunan, China; 7 State Key Laboratory of Oncology in South China, Collaborative Innovation Center for Cancer Medicine, Sun Yat-Sen University Cancer Center, Guangzhou 510060, Guangdong, China

**Keywords:** *TILRR*, immune cell infiltrating, prognostic biomarker, breast cancer

## Abstract

In atherosclerosis, upregulated TILRR (FREM1 isoform 2) expression increases immune cell infiltration. We hypothesized that *TILRR* expression is also correlated with cancer progression. By analyzing data from Oncomine and the Tumor Immune Estimation Resource, we found that *TILRR* mRNA expression was significantly lower in breast cancer tissue than adjacent normal tissue. Kaplan-Meier survival analysis and immunohistochemical staining revealed shortened overall survival and disease-free survival in patients with low TILRR expression. *TILRR* transcript expression was positively correlated with immune score, immune cell biomarkers and the expression of *CXCL10* and *CXCL11*. *TILRR* expression was also positively correlated with CD8+ and CD4+ T-cell infiltration. These correlations were verified using the ESTIMATE algorithm, gene set enrichment analysis and Q-PCR. We concluded that impaired *TILRR* expression is correlated with breast cancer prognosis and immune cell infiltration.

## INTRODUCTION

The product of the FRAS1-related extracellular matrix 1 (*FREM1*) gene was first identified as a secretory protein excreted by mesenchymal cells that play a critical role in the development of multiple organs [1]. Multiple *FREM1* transcripts can be found in the mammalian system [2]. In a previous study, TILRR (Toll-like/IL-1 receptor regulator) was identified as the IL-1R co-receptor, a 715-amino acid heparan sulfate glycoprotein encoded within the gene for the extracellular matrix protein FREM1 [2]. Hence, in the National Center for Biotechnology database, TILRR is annotated as FREM1 (isoform 2).

TILRR binds to the cell membrane through a C-terminal lectin domain, and partners with IL-1R1 as its co-receptor to enhance ligand binding. Overexpressed TILRR interacts with IL-1R1 via its TIR domain. This association potentiates the recruitment of the MyD88 adapter protein, and the signal amplification enhances activation of NF-ĸB and pro-inflammatory genes [3].

Recently, it was reported that TILRR upregulates pro-inflammatory gene expression in the progression of atherosclerosis [4]. Because TILRR induces immune cell infiltration, we wondered if TILRR expression might contribute to tumor progression. We hypothesized that the influence of TILRR on pro-inflammatory gene expression might have a prognostic value in cancer treatment.

First, we examined the correlation between FREM1 expression and the prognosis of cancer patients. Subsequently, we investigated the expression of TILRR in tumor cells within different tumor microenvironments. The findings shed light on the crucial role of TILRR in breast cancers. The protein may be useful as a prognostic biomarker.

## RESULTS

### *FREM1* mRNA expression levels in human breast cancer tumors

**Figure 1:**
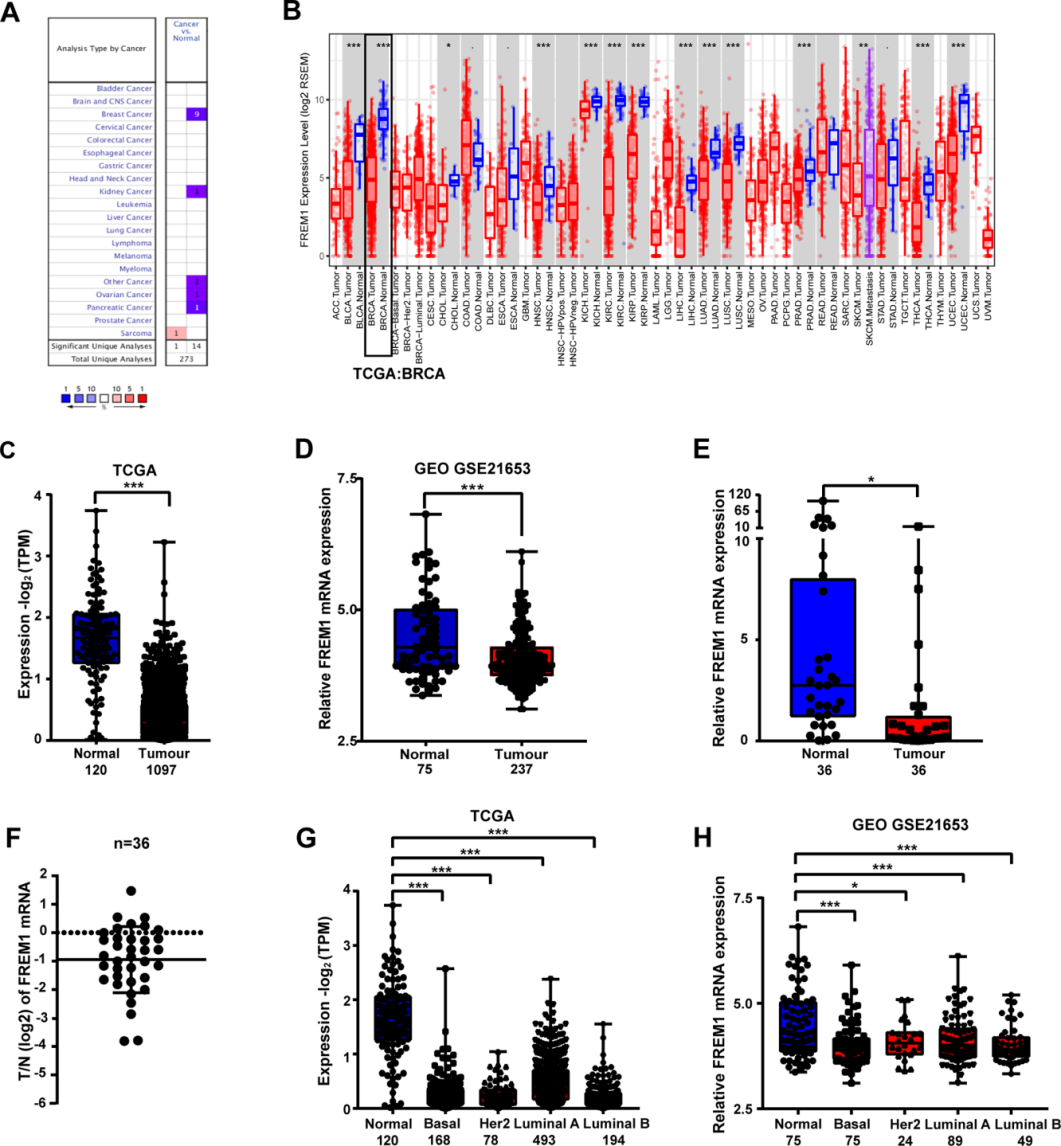
***FREM1* expression levels in different types of human cancers.** (**A**) Expression levels of *FREM1* in different types of cancer compared with normal tissues. Data is from the Oncomine database. (**B**) Expression levels of *FREM1* in different tumor types from the TCGA database were analyzed by TIMER. (**C**, **D**) *FREM1* expression levels analyze by TIMER in normal and tumor samples from the TCGA and GEO databases. (**E**, **F**) *FREM1* expression levels were analyzed by Q-PCR in paired normal and tumor tissues. (**G**, **H**) *FREM1* expression levels in normal and subtypes of breast cancer samples from the TCGA and GEO databases were analyzed by TIMER. (*P <0.05, ** *P* < 0.01, ***P < 0.001).

To investigate the role of *FREM1* gene expression in cancer, we determined the *FREM1* mRNA levels in tumor and normal adjacent tissues of 20 cancer types. Data obtained from Oncomine was analyzed using the following threshold criteria: 2-fold change, *P* value < 0.0001 and a gene rank of 10%. Downregulation of *FREM1* gene expression was found in breast cancer, ovarian cancer and pancreatic cancer tissues. In breast cancer tissues, 9 out of 43 samples met the threshold criteria in 4 out of 10 datasets. In the other two cancer types, only a single event reached threshold criteria (Figure 1A).

Further evaluation of *FREM1* expression in multiple malignancies was carried out using the gene expression data from The Cancer Genome Atlas (TCGA). Differential *FREM1* expression between the tumor and adjacent normal tissues is illustrated in Figure 1B. In most types of cancer, *FREM1* expression is significantly lower (*P* value < 0.001) than in adjacent normal tissues (Figure 1B).

*FREM1* expression in breast cancer was further investigated using TCGA and Gene Expression Omnibus (GEO) data (Figure 1C and 1D). *FREM1* expression in tumor tissues was 1.25-fold (GEO) and 4.18-fold (TCGA) lower than in normal adjacent tissues. These findings were confirmed in paired patient biopsy samples via Q-PCR (N = 36, *P* = 0.0336) (Figure 1D and 1E). In tumor biopsy samples, *FREM1* expression was reduced by as much as 6.16-fold (*P* < 0.001). In 17 samples (47.2%), expression was decreased more than 2-fold (Figure 1E). We expanded our analysis of *FREM1* expression to various subtypes of breast cancer (Figure 1G and 1H). This analysis compared normal tissue (n = 120/n = 75) with tissues from the following breast cancer subtypes: Basal-like (n = 168/n = 75), *HER2*-enriched (n = 78/n=24), Luminal-like A (n = 493/n = 89) and Luminal-like B (n = 194/n = 49). These data showed decreased *FREM1* expression in Basal-like (6.07-fold/1.11-fold with mean), *HER2*-enriched [6.79-fold/1.09-fold with mean (*P* = 0.0279)], Luminal-like A (3.46-fold/1.08-fold with mean) and Luminal-like B (7.54-fold/1.11-fold with mean) tissues. Hence, *FREM1* expression is downregulated in breast cancer tissue.

### *FREM1* transcription level is correlated with survival and progression in breast cancer patients

**Figure 2:**
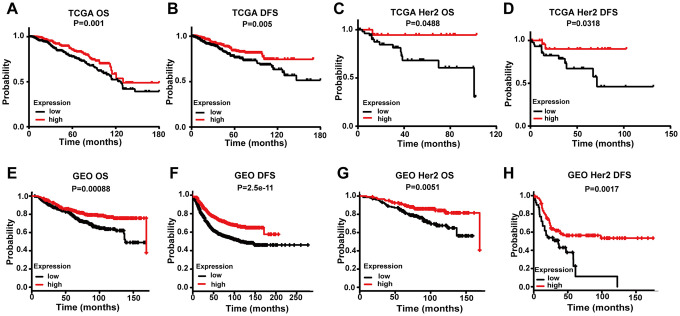
**Comparing the low and high expression levels of *FREM1* by Kaplan-Meier survival analysis in breast cancer and the *HER2* subtype.** (**A**–**D**) Survival curves of OS and DFS in breast cancer and *HER2* from the TCGA database. (**E**–**H**) Survival curves of OS and DFS in breast cancer and *HER2* from the GEO database. OS, overall survival; DFS, disease-free survival.

We further analyzed TGCA data to determine if there is a relationship between *FREM1* expression and overall survival (OS) or disease-free survival (DFS) in breast cancer patients. Patients were divided into high- or low-level groups over the median value of *FREM1* expression in breast cancer tissues. Patients with high *FREM1* expression in their tumors experienced a prolonged OS and DFS compared with those who with low *FREM1* expression (Figures 2A and 2B). Additionally, KM analysis revealed that *HER2*-positive *BRCA* patients in the low-level *FREM1* expression group generally demonstrated shorter DFS (Figures 2C and 2D). To confirm the clinical significance of downregulated *FREM1* expression in breast cancer patients, the correlation between survival rate and *FREM1* transcription was determined. Survival analysis of GEO data indicated that low-level *FREM1* expression was correlated with reduced OS and DFS (Figure 2E and 2F), and the same scenario was shown in *HER2*-positive *BRCA* patients (Figure 2G and 2H). This data was consistent with the results obtained from TCGA cohort. These results suggest that reduced *FREM1* transcription influences breast cancer tumor progression and is associated with shortened patient survival.

### Downregulation of the FREM1 protein in primary human breast cancer tissues correlates with disease progression

**Figure 3:**
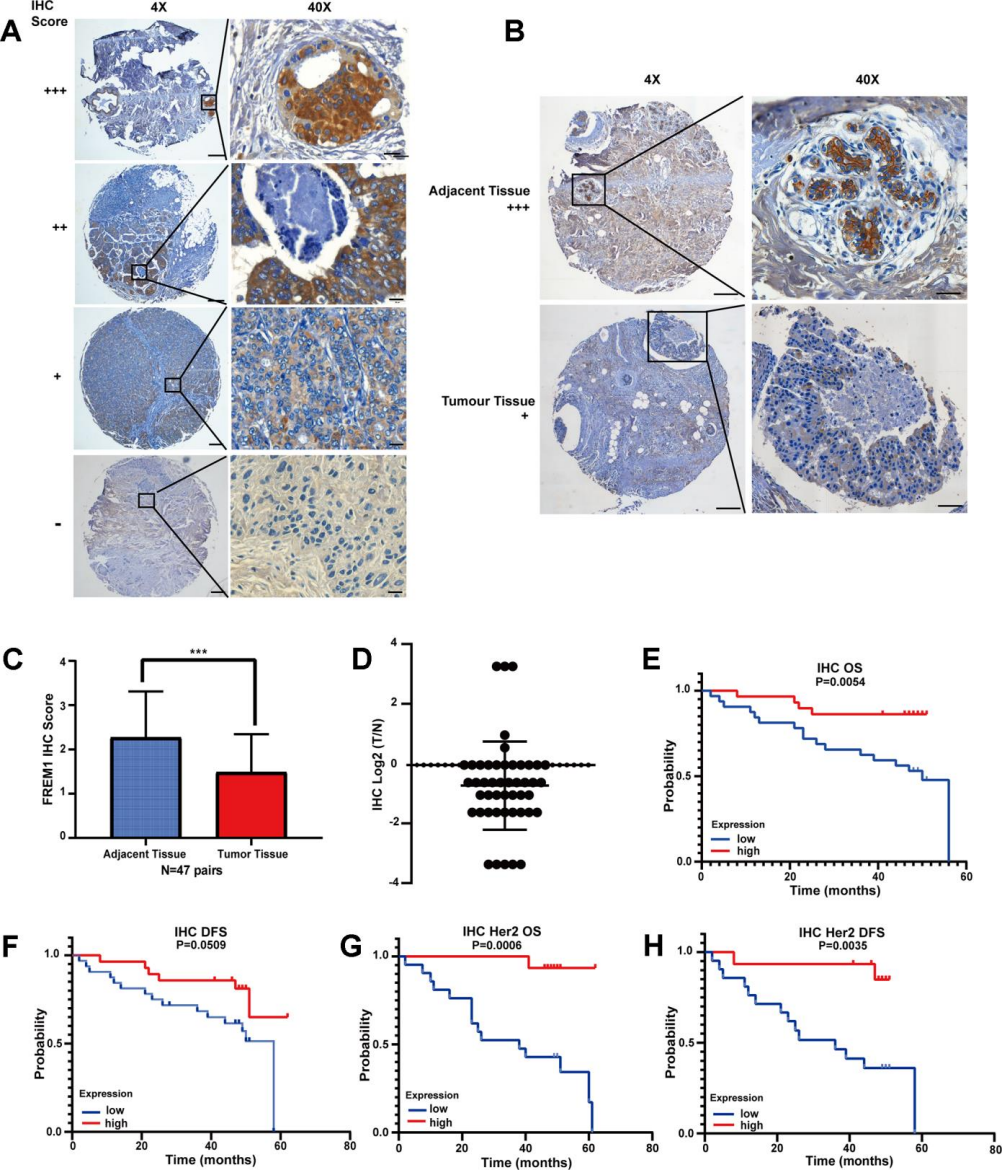
**FREM1 expression is reduced in tumor tissues, which is associated with poor survival.** (**A**) Representative IHC staining of FREM1 in human primary tumor and adjacent non-tumor tissue. Magnifications: 4X; boxed area is 40X. Scale bar: 4X, 200 μm; 40X, 20 μm. (**B**) FREM1 level is higher in adjacent tissues than in tumor tissues. Magnifications: 4X; and boxed area is 40X. Scale bar: 4X, 200 μm; 40X, 20 μm. (**C**, **D**) Analysis of FREM1 IHC staining scores in adjacent non-tumor (n = 48) and tumor tissue (n = 56). (*P < 0.05, **P < 0.01, ***P < 0.001). (**E**–**H**) Survival curves of OS and DFS in breast cancer and *HER2* with low and high *FREM1* expression. Median survival time of the high-expression group versus low-expression group.

To determine the clinical significance of the FREM1 protein, we performed representative immunohistochemical (IHC) staining in primary human breast cancer tumor and adjacent normal tissue (N = 47). FREM1 staining in human primary breast cancer was scored as follows: +++, high; ++, moderate; +, weak; −, negative. FREM1 expression was significantly decreased in tumors, as indicated by lower IHC staining scores (*P* < 0.001) (Figure 3A and 3B). Among the 47 breast cancer samples, FREM1 staining was high in 4 samples (8.5%), moderate in 22 samples (46.8%) and weak or undetectable in 21 samples (44.7%) (Figure 3C and 3D). The AOD (average optical density) quantified data set was plotted in Supplementary Figure 1.

From the clinical information provided with the tissue chip, the survival rate was analyzed. High FREM1 expression correlated with a prolonged OS and DFS compared with low FREM1 expression, but the statistical significance of the DFS prolongation was weak (*P* = 0.0509) (Figure 3E and 3F). When data was re-analyzed only using *HER2*-enriched samples, the correlation between FREM1 expression and survival rate was robustly significant in both OS (*P* = 0.0006) and DFS (*P* = 0.0035) (Figure 3G and 3H). Thus, FREM1 downregulation in breast cancer tissue, especially in *HER2*-enriched samples, was correlated with shorter survival times. This finding from IHC staining is consistent with our database analysis.

### *TILRR* is the clinically relevant isoform of *FREM1* in breast cancer

**Figure 4:**
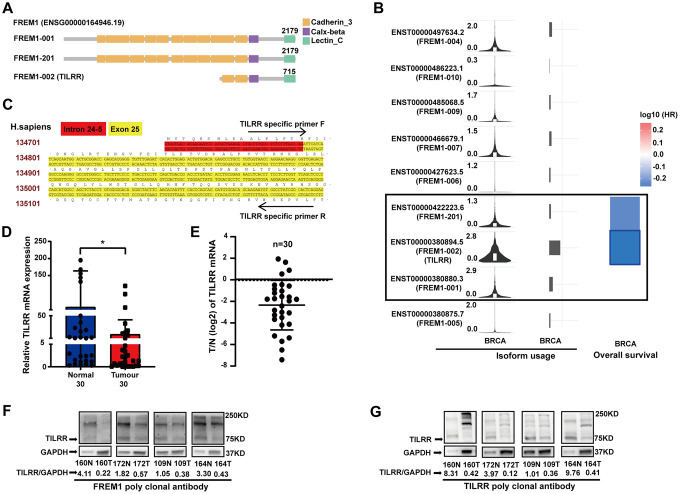
***TILRR*, the *FREM1* isoform 2 transcript, is clinically relevant in breast cancer.** (**A**) The three *FREM1* isoforms. (**B**) *TILRR*, the gene encoding isoform 2, shows convincing cancer specificity with strong usage and significantly low hazard ratio. (**C**) *TILRR*-specific primer of human DNA. (**D**, **E**) *TILRR* expression levels were analyzed by Q-PCR in paired normal and tumor tissues (*P < 0.05, **P < 0.01, ***P < 0.001). (**F**, **G**) TILRR immunoblotting (indicated by the *FREM1* or *TILRR* antibody) in paired normal and tumor tissues. The band intensities are normalized to GAPDH.

Previously, *TILRR* has been identified as isoform 2 of the *FREM1* gene [3]. We wanted to determine if *TILRR* is the dominate isoform that is downregulated in breast cancer. In the Ensembl and Gene Expression Profiling Interactive Analysis 2 (GEPIA2) databases, three different *FREM1* isoforms are described. In these isoforms, ENST00000422223.6 and ENST00000380880.3 encode a protein with 2179 amino acids, and the much shorter version of ENST00000380894.5 encodes a protein with 715 amino acids. ENST00000380894.5 is the FREM1 isoform 2 transcript, which we referred to previously as *TILRR* (Figure 4A). The correlation between breast cancer survival rate and *FREM1* usage was investigated using the GEPIA2 database. In Figure 4B, the violin-plot and bar-plot panels present the expression distribution and usage of each *FREM1* isoform. TILRR, or FREM1 isoform 2, shows convincing cancer specificity with strong usage and a significantly low hazard ratio (HR).

Using a *TILRR*-specific primer (Figure 4C), *TILRR* expression in 30 paired breast cancer samples was investigated using Q-PCR. The data showed a 4.39-fold decrease (*P* = 0.0274) in expression in tumor tissue compared with normal tissue (Figure 4D and 4E), which confirmed that *TILRR* is downregulated in breast cancer tissues. The protein expression identity of *FREM1* and *TILRR* was verified using four paired breast cancer tissues (sample numbers: 160, 172, 109 and 164) (Figure 4F and 4G). The *FREM1* commercial polyclonal antibody blotting pattern was identical to that of the TILRR polyclonal antibody, which detected a 75 KDa peptide with an approximately 4.76- and 9.09-fold reduction in expression in tumor tissues. A similar finding was discovered using TILRR monoclonal probes (data not shown). These results confirm that downregulation of *TILRR* is associated with breast cancer prognosis.

### Analysis of *TILRR* transcript level associated with tumor-infiltrating lymphocytes

**Figure 5:**
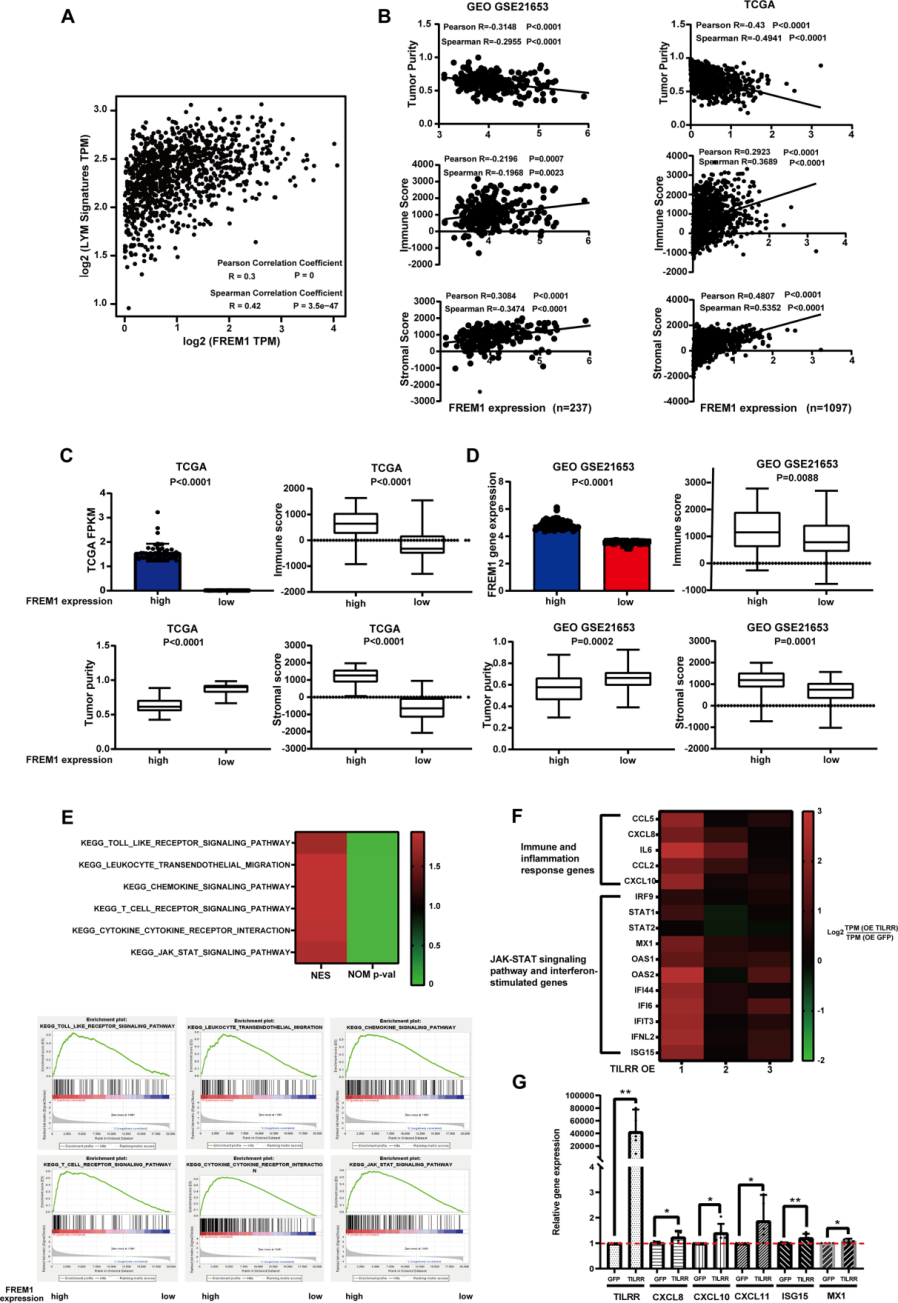
**FREM1 mRNA levels were associated with tumor infiltrating lymphocytes.** (**A**) The average expression of the LYM metagene signature [*PTPRC (CD45), CD53, LCP2 (SLP-76), LAPTM5, DOCK2, IL10RA, CYBB and CD48, ITGB2 (LFA-1)* and *EVI2B*] in breast cancers from the TCGA database relative to the *FREM1* mRNA level. (**B**–**D**) Tumor purity, immune score and stromal score were analyzed using the ESTIMATE algorithm from GEO (N = 237) and TCGA (N = 1097) database. (**E**) GSEA analysis was used to demonstrate the correlation between *FREM1* expression and the KEGG enriched pathway. (**F**) Immune and inflammation response genes, the JAK-STAT signaling pathway and interferon-stimulated genes were analyzed by RNA-seq in BT474 cancer cells. (**G**) *CXCL8*, *CXCL10*, *CXCL11* and the interferon stimulation genes, *ISG15* and *MX1*, were analyzed by Q-PCR in BT474 cancer cells.

As shown above, *TILRR* is the dominant isoform of *FREM1* expressed in breast cancer tissues. A prior study found that TILRR is related to monocyte infiltration in atherosclerosis plaque development [4]. Moreover, the lymphocyte-specific immune (LYM) recruitment metagene signature is related to tumor infiltration by lymphocytes, and it is associated with a favorable prognosis in breast cancer. To investigate the role of TILRR in the mediation of immune cell infiltration, the correlation between *TILRR* expression and LYM was evaluated in TCGA breast cancer data. The LYM metagene sets *PTPRC* (*CD45*), *CD53*, *LCP2* (*SLP-76*), *LAPTM5*, *DOCK2*, *IL10RA*, *CYBB,* *CD48*, *ITGB2* (*LFA-1*) and *EVI2B* were positively correlated with expression of *FREM1* (Spearman *r* = 0.42 *P* = 3.5e-47; Pearson *r* = 0.3 *P* = 0) (Figure 5A).

The estimation of stromal and immune cells in malignant tumor tissues using expression data(ESTIMATE) algorithm can calculate the gene expression signature in tumor cells and normal, tumor-associated epithelial and stromal cells, immune cells and vascular cells [5]. Stromal cells are thought to have essential roles in tumor growth, disease progression and drug resistance, and ESTIMATE immune scores could serve as an indicator for immunotherapy response [5]. Tumor purity and the expression of *TILRR* were analyzed using the ESTIMATE algorithm to investigate in GEO (N = 237) and TCGA (N = 1097) data. The results showed that the expression of *TILRR* was negatively correlated with tumor purity, but positively correlated with immune score and stromal score (Figure 5B). This correlation was confirmed with the 50 highest and 50 lowest TILRR-expressing tumor tissue samples (Figure 5C and 5D).

To understand the underlying mechanism of *FREM1* in breast cancer, we analyzed differentially expressed data from the 50 highest and 50 lowest *FREM1*-expressing tumor tissue samples. The gene expression enrichment of several signal pathways was analyzed using gene set enrichment analysis (GSEA). *TILRR* is positively correlated with Toll-like receptor signaling and cytokine-to-cytokine receptor interactions (Figure 5E), which is consistent with our previous finding [3]. We also found that leukocyte transendothelial migration, chemokine signaling, T-cell receptor signaling and JAK-STAT signaling are associated with the migration and infiltration of immune cells (Figure 5E). To verify these pathways, RNA-seq analysis of the BT474 cancer cell line overexpressing *TILRR* was performed using an empty GFP construct as a control. The analysis showed that *TILRR* overexpression not only potentiates *IL-6* and *CXCL8* immune- and inflammation-induced gene expression, but it also induces the expression of other cytokine genes such as *CCL5/2* and chemokine genes such as *CXCL10* (Figure 5F). JAK-STAT signaling pathways and interferon-stimulated genes (further detailed analysis will be published elsewhere) were also upregulated (Figure 5F). This upregulation of pro-inflammatory secretory factors and pathway activation indicated that enhanced *TILRR* expression might cause long distal immune recruitment. Q-PCR was performed to confirm RNA-seq detection among expression of *CXCL8, CXCL10, CXCL11,* *MX1* and the interferon stimulation gene, *ISG15* (Figure 5G).

Furthermore, we analyzed the 50 highest *TILRR*-expressing and 50 lowest *TILRR*-expressing tumor tissue samples (TCGA database) to determine the relationship between the expression of *CXCL10* and *CXCL11* and the expression of *TILRR* in cancer tissues. The results showed that high expression of *TILRR* positively correlates with *CXCL10* and *CXCL11* expression (Supplementary Figure 2). These findings strengthened the hypothesis that *TILRR* expression is associated with breast cancer progression and prognosis, likely through signaling pathways that regulate the distal recruitment of immune cell infiltration.

### Expression of *TILRR* is correlated with immune cell infiltration in breast cancer

**Figure 6:**
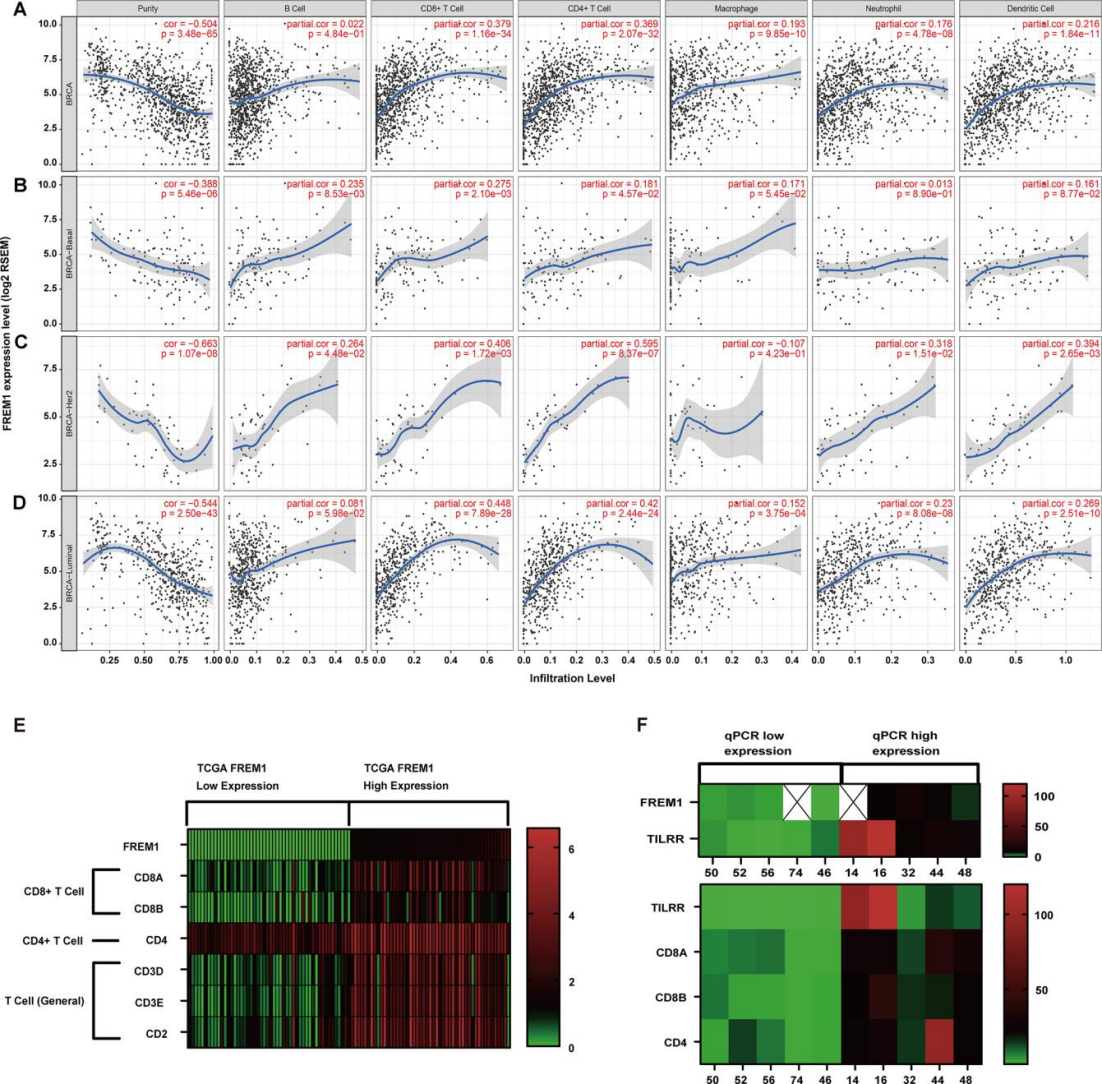
**Correlation of *FREM1* expression with immune infiltration level in the subtypes of breast cancer.** (**A**) *FREM1* expression is negatively related to tumor purity and has significant positive correlations with infiltrating levels of B cells, CD8+ T cells, CD4+ T cells, macrophages, neutrophils and dendritic cells in *BRCA*. (**B**) *FREM1* expression is negatively related to tumor purity and has positive correlations with infiltrating levels of B cells and CD8+ T cells, but not CD4+ T cells, macrophages, neutrophils and dendritic cells in *BRCA*-Basal. (**C**) FREM1 expression is significantly negatively related to tumor purity and has significant positive correlations with infiltrating levels of CD8+ T cells, CD4+ T cells and dendritic cells, but not B cells, macrophages and neutrophils in *BRCA*-*HER2*. (**D**) FREM1 expression is negatively related to tumor purity and has significant positive correlations with infiltrating levels of CD8+ T cells, CD4+ T cells, macrophages, neutrophils and dendritic cells, but not B cells in *BRCA*-Luminal. (**E**) The top 50 highest TILRR-expressing and top 50 lowest *TILRR*-expressing tumor tissue samples were selected (TCGA database) to analyze the expression of marker genes in CD8+, CD4+ and T cells (general). (**F**) *CD8A, CD8B* and *CD4* expression levels were analyzed by Q-PCR in the 5 highest *TILRR* expressing and the 5 lowest *TILRR* expressions samples.

Increasing evidence suggests that tumor-infiltrating immune cells can be an indicator in the clinical analysis of tumor samples [6, 7]. Gene expression profiling of heterogeneous cell populations in cancer tissue, including tumor-infiltrating lymphocytes, serves as an independent predictor of survival in prognostic cancer models [8, 9]. Thus, we used the TIMER database to evaluate the correlation between *FREM1* mRNA expression and six different infiltrating immune cell types (B cells, CD8+ T cells, CD4+ T cells, macrophages, neutrophils and dendritic cells) in different subtypes of breast cancer. Data showed that the TILRR transcription level was inversely related to the purity of tumor tissue in breast cancer subtypes (Figure 6A–6D, first line on the left). The low heterogeneity in tumor tissue correlated with high expression of *FREM1* in all but one subtype, which is consistent with the positive association between *TILRR* expression and level of immune cell infiltration (Figure 6). The exception was macrophage cells of the *HER2* subtype (*r* = -0.107, *P* = 4.23e-01). The infiltration levels of CD8+ and CD4+ cells were significantly positively correlated with *TILRR* transcription. The level of CD8+ T-cell infiltration with *TILRR* expression in *BRCA* (*r* = 0.379, *P* = 16e-34), *BRCA*-Basel (*r* = 0.275, *P* = 2.10e-03), and *BRCA-HER2* (*r* = 0.406, *P* = 1.2e-03), and *BRCA*-Luminal (*r* = 0.48, *P* = 7.89e-28) were all significantly strong, respectively, which is similar to the CD4+ T-cell infiltration levels (*BRCA*: *r* = 0.369, *P* = 2.07e-32; Basal: *r* = 0.181, *P* = 4.57e-02; *HER2*: *r* = 0.595, *P* = 8.37e-07; luminal: *r* = 0.42, *P* = 2.44-24e). On the other hand, in the tumor tissue, the infiltration level from B cells, macrophages, neutrophils and dendritic cells correlated more weakly with *TILRR* transcription in subtypes of breast cancer (Figure 6). To verify the expression of *TILRR* and the infiltration of immune cells, the 50 highest TILRR-expressing and 50 lowest TILRR-expressing tumor tissue samples were selected (TCGA database) to analyze the expression of marker genes for CD8+, CD4+ and T cells (general). The results showed that *CD8A*, *CD8B* was expressed in tissues with high *TILRR* expression. (Figure 6E). Analysis of GEO data generated similar results (Supplementary Figure 3). To further verify this result, we selected the 5 samples with the highest *TILRR* expression and the 5 with the lowest *TILRR* expression patients shown in Figure 4D to evaluate the expression of marker genes in CD4- and CD8-expressing cells. Among these 10 samples, the expression of *CD8A*, *CD8B* and *CD4* in *TILRR* high- and low-expressing samples were positively correlated (Figure 6F). We also investigated the correlation between *TILRR* expression and biomarkers from other immune cells. Strong associations were found between NK cells, Treg cells, T-cell exhaustion and *TILRR* expression (Supplementary Table 1). Consequently, it is likely that *TILRR* expression in breast cancer tissue is involved in immune cell recruitment.

## DISCUSSION

In the present study, we observed that *TILRR* expression level was associated with the survival of patients in breast cancer. Database analysis revealed that *TILRR* mRNA expression was significantly lower in breast cancer tissue and correlated with shorter OS and DFS. The TCGA and GEO data analysis were confirmed by IHC staining and Q-PCR. *TILRR* transcript expression is correlated with immune score, immune cell biomarkers and LYM metagene signature, which was verified using the ESTIMATE algorithm and GEPIA2 database analysis. This consistent association between increased *TILRR* mRNA levels and a favorable prognosis for the patient is shown in Figure 4. Two of five patients with higher *TILRR* mRNA levels had a favorable prognosis (Figure 6F). However, in the group with lower *TILRR* mRNA, 3 out of 5 patients were dead due to cancer (data not shown). To our knowledge, this is the first study to describe *TILRR* expression in cancer tissue.

In this study, we found that TILRR expression is profoundly downregulated in breast cancer and correlated with disease-specific survival. The PyMT mouse, a genetically engineered mouse model that is widely used to study human breast cancer, gene profiling and expression analysis has illustrated the effects of *TILRR* downregulation [10]. In *PyMT/ Il1a-/-* and *PyMT/Il1r1-/-* mice, IL-1R1 signaling suppresses mammary tumor cell proliferation early in tumorigenesis and facilitates breast cancer outgrowth with pulmonary metastasis [11]. IL-1Ra is overexpressed in multiple cancers, including multiple myeloma, leukemia, cervical, ovarian, colorectal, pancreatic and breast cancer, but is downregulated in others [11–15]. This is consistent with the idea that *TILRR* enhances IL-1 alpha affinity binding to IL-1R1, as IL-1R1 is a low-copy, high-affinity receptor [3, 16]. Interestingly, the IL-1R1 signaling pathway has been reported to promote tumor growth, angiogenesis and metastasis in some contexts [17], while stimulating anti-tumor immunity or directly suppressing tumorigenesis in others [11]. TILRR activates oncogene *RAS* upstream of *TRAF6* in the IL-1R1-mediated pathway, and *TILRR* overexpression enhances AKT phosphorylation and HeLa-cell survival via the *TILRR* R425 site [18]. Previously it was reported that under *LDLR-/-* or *APOE-/-* genetic conditions, monocyte activation and infiltration were reduced in atherosclerosis and in the lung of *TILRR* KO mice or mice injected with *TILRR*-blocking antibody [4]. As reported, under *LDLR-/-* conditions, mouse models of hypercholesterolemia developed a smaller tumor. These mice were characterized by increased LDLR expression, as well as shorter OS and decreased DFS [19]. Interestingly, *TILRR* expression level is low in healthy tissue; however, it is remarkably enhanced in atherosclerotic plaques with a high level of immune cell infiltration [4].

Another important aspect of this study is the correlation between *TILRR* expression and the level of immune infiltration. Through data analysis, we observed associations between *TILRR* mRNA levels and immune score, immune cell biomarkers, the LYM metagene signature and levels of infiltrating immune cells. In previous studies, *TILRR* expression was correlated with monocyte infiltration; notably, *TILRR*-/- mice showed less recruitment of immune cells in atherosclerosis plaques [4]. ESTIMATE calculation and GSEA analysis of the top 50 highest *TILRR*-expressing samples compared with the top 50 lowest samples showed that *TILRR* transcription played a role in immune cell infiltration, migration and activation. On the other hand, RNA-seq and Q-PCR analysis showed that *TILRR* potentiates *CXCL10* and *CXCL11* chemokine expression in the BT474 cancer cell line. In response to specific chemokines, immune cells can regulate immune responses by migrating into the tumor microenvironment. It has been reported that the tumor production of *CXCL9* and *CXCL10* was repressed by enhancement of H3K27me3 and DNMT1-mediated DNA methylation. Moreover, EZH2 and DNMT1 are negatively associated with tumor-infiltrating CD8+ T cells [20]. In the tumor microenvironment, *CXCL11* upregulation enhanced CD8+ T-cell recruitment [21]. Collectively, we speculate that, in the tumor microenvironment, *TILRR* can enhance immune infiltration by regulating the *CXCL10* and *CXCL11* chemokines.

It has been confirmed that IL-18 potentiates IFN-γ-induced *CXCL9*, *CXCL10*, and *CXCL11* mRNA expression and secretion by activating the NF-ĸB and JAK-STAT signaling pathways [22]. Our previous studies showed that *TILRR* overexpression can increase the activation of the NF-ĸB signaling pathway, which agree with our RNA-seq data in this study. Interestingly, we also observed IFN-γ-induced gene expression; however, whether the *TILRR* effect on the upregulation of *CXCL-10* and *CXCL*-11 was through an indirect cytokine chain reaction of multiple cell types remains unknown. We found in these tissues a positive correlation between *TILRR* upregulation and IFN-γ-related signaling pathways. These results proved that the *TILRR* transcription level could indicate lymphocyte infiltration in breast cancer.

*TILRR*-related immune cell recruitment in breast cancer showed the strongest correlation with CD8+ positive cells and the T cell-related pathway. In cancer treatment, inhibiting immune checkpoint mediators, such as CTLA-4 and PD-1, has achieved noteworthy clinical outcomes in several malignancies [23–26]. Cardiovascular disease is also the consequence of targeted cancer therapies and chemotherapies in several clinical settings [27, 28]. *TILRR* expression related to chemokine secretion and NF-ĸB activation is well documented in cardiovascular disease [4]. Therefore, we conclude that *TILRR* might recruit immune cells through a similar cellular mechanism in breast cancer and atherosclerosis. We believe that *TILRR* could play different roles in different organs but may share a similar mechanism in breast cancer and atherosclerosis.

## MATERIALS AND METHODS

### *FREM1* gene expression of *BRCA* in the TCGA and GEO databases

The expression level of the *FREM1* gene in various types of cancers was identified in the Oncomine database (https://www.oncomine.org/resource/login.html) [29]. The threshold was determined according to the following values: *P* value of 0.001, fold change of 2. Differential expression module of TIMER (https://cistrome.shinyapps.io/timer/) was used to analyze the *FREM1* differential expression between tumor and adjacent normal tissues of various TCGA tumors [30, 31]. TCGA breast cancer data was downloaded from the Xena browser (https://xenabrowser.net/datapages/) [32]. GSE21653 data was downloaded from the GEO database, which contained 266 early cancer patients [33, 34].

### Immune cell infiltration analysis

The correlation of *FREM1* expression level with a LYM metagene signature [*PTPRC (CD45), CD53, LCP2 (SLP76), LAPTM5, DOCK2, IL10RA, CYBB, CD48, ITGB2 (LFA-1)* and *EVI2B*] was explored via Gene Expression Profile Interactive Analysis (GEPIA2; http://gepia2.cancer-pku.cn/#index) [35–37]. The stromal score, immune score and tumor purity of breast cancer (*BRCA*) tumor samples from the GSE21653 database (N = 237) and the TCGA database (N = 1097) were calculated by using the R 3.6.0 ESTIMATE package (1.0.13) [38]. The gene module of TIMER was used to evaluate the correlation of *FERM1* expression with immune cell infiltration (including B cells, CD4+ T cells, CD8+ T cells, neutrophils, macrophages and dendritic cells) in *BRCA*, which determined the purity-corrected partial Spearman’s correlation and statistical significance. The correlation module of TIMER was used to calculate the Spearman’s correlation and statistical significance between *FREM1* and immune cell marker genes. The top 50 highest and lowest *FREM1*-expressing TCGA tumor samples were used to conduct GSEA and Kyoto Encyclopedia of Genes and Genomes (KEGG) pathway annotations analysis [39].

### RNA isolation and real-time PCR

Total mRNA of breast cancer tissue (SYSUCC, Sun Yat-sen University Cancer Center, Guangzhou, China) was extracted using TRIzol reagent (Invitrogen, #1556018) according to the manufacturer’s protocols. The quantitative, real-time PCR using SsoAdvanced^TM^ Universal SYBR Green Supermix (Bio-Rad, #1725274) was performed in an ABI StepOnePlus (ABI, #1725274). *FREM1* primer sequences: forward primer, 5’-AGAGCCCTGCCTGTGGTAAC-3’; reverse primer, 5’-GAAGGGGAATGCAAGAGTGTGATA-3’. *TILRR*-specific primer 5’-GCCTTGCCTCTCTTTACCAGAT-3’; reverse primer, 5’-GAGTGCCGATAGGCCACAT-3’. Relative gene expression was normalized to glyceraldehyde 3-phosphate dehydrogenase (*GAPDH)* (forward primer, 5’-GGAGCGAGATCCCTCCAAAAT-3’; reverse primer, 5’-GGCTGTTGTCATACTTCTCATGG-3’) expression and was analyzed using the 2^−ΔΔCT^ method.

### Immunohistochemistry (IHC)

Immunohistochemistry was performed on tissue section microarrays (Zhuolibiotech, #ZL-Brc Sur122). The staining procedure included heat-induced epitope retrieval using 0.1 M sodium citrate (pH 9.0, heated by microwave to 90-95 °C, 3 times for 5 minutes each), incubation with primary antibody at 4 °C overnight. Signal detection was performed using an IHC detection kit (Gene Tech, #GK500710). Microscopy of the immunostaining included an initial pre-screen at low power (4X) to identify regions with a technically optimal staining result. Subsequently, detailed analysis at high-power (40X) was performed to evaluate the staining according to routine algorithms employed in tumor diagnostics. Scoring of FREM1 staining were evaluated using the semi-quantitative immunostaining score (ISS) method by pathologist. The immunostaining score was defined as 0 – 3 (range: +++/3, high; ++/2, moderate; +/1, weak; 0, negative.) [40]. The median score was used as cut off for classification of patients into high- and low- expression groups. Semi-quantitative analysis of the IHC images was conducted by Image-J, by which integral optical density (IOD) and the area were collected. Then, average optical density (AOD) was calculated as IOD/area, which represented the staining intensity [41].

### Western blot

Total proteins were extracted from breast cancer and adjacent cancer tissue (SYSUCC, Sun Yat-sen University Cancer Center, Guangzhou, China) by using RIPA buffer (Beyotime, #P0013B) and passed the tissue through a 21-gauge needle more than 30 times. The cell lysate was centrifuged at 4 °C and 17000 g for 15 minutes to eliminate large aggregates. A BCA Protein Assay Kit (Tiangen, #PA115) was used to evaluate the cell lysate protein concentrations. Approximately 25-50 μg total protein, denatured with the standard SDS-sample buffer per lane, was loaded for electrophoresis on 10% pre-cast mini-polyacrylamide gels (GenScript SurePAGE, Bis-Tris, 10 cm x 8 cm gels). The gel containing proteins was then transferred to a PVDF membrane (BioRad). The membrane was blocked with 5% milk in 1×TBST for 2 hours, and incubated with the FREM1 primary antibody (1:1000 rabbit polyclonal, Proteintech, #13086-1-AP) and *TILRR* (1:2000 rabbit polyclonal custom, Genesript) at 4 °C overnight. Before incubating the membrane with a secondary antibody (1:10000 dilution, Jackson ImmunoResearch, #111-035-003) for 1 hour, it was washed the with 1×PBST 5 times at room temperature. The blots were detected by chemiluminescence (Bio-Rad). The band intensity was quantified by ImageJ (https://imagej.nih.gov/ij/download.html).

### Cell culture

Cells were cultured (BT474, purchased from Procell Life Science and Technology Co., Ltd. CL-0040) at the density of 1×10^6^/well in RPMI 1640 Medium (Gibco, #C11875500BT) with 20% FBS and insulin. Cells were incubated at 37 °C and 5% CO_2_. The day after seeding, cells were transfected with HA-GFP or HA-*TILRR*-T2A-GFP plasmid using the Lipofectamine 3000 Transfection Reagent (Invitrogen, #L3000-015). Cells were incubated at 37 °C and 5% CO_2_ for 24 hours, at which time culture medium was replaced. Forty-eight hours after transfection, cells were detached by 0.25% trypsin for 3 minutes, and then collect the cell for cell sorting (Flow Cytometer, Aria II, BD).

### RNA-seq

Trizol lysate was used to lyse selective cells and extract RNA from the cells. To generate sequencing libraries, 1 ug RNA per sample was used (NEBNext UltraTM RNA Library Prep Kit, Illumina; NE, USA). Library quality was assessed on the Agilent Bioanalyzer 2100 system. Sequencing was performed on the Illumina Novaseq platform, and 150 bp paired-end reads were generated. Raw reads were aligned to a reference genome (UCSC GRCh38/hg38) using Bowtie 2 (2.2.5). Gene expression was quantified using RSEM v1.1.22.

### Statistical analysis

OS and DFS curves were generated by Kaplan-Meier survival analysis using SPSS 17.0 software. The results generated in Oncomine are displayed with *P* values, fold changes, and ranks. The results of the Kaplan-Meier plots, PrognoScan, and GEPIA2 are displayed with HR and *P* or Cox *P* values from a log-rank test. The correlation of gene expression was evaluated by Spearman’s correlation and statistical significance, and the strength of the correlation was determined using the following guide for the absolute value: 0.00–0.19, very weak; 0.20–0.39, weak; 0.40–0.59, moderate; 0.60–0.79, strong; 0.80–1.0, very strong. *P* values < 0.05 were considered as statistically significant.

## Supplementary Material

Supplementary Figures

Supplementary Table 1
