# Supplementary material for: TILRR (FREM1 isoform 2) is a prognostic biomarker correlated with immune infiltration in breast cancer
Source: Aging (Albany NY). 2020 Oct 8;12(19):19335–51. doi: 10.18632/aging.103798 (PMC7732299; doi:10.18632/aging.103798)
Supplement: Supplementary Figures [file aging-12-103798-s001..pdf]

SUPPLEMENTARY FIGURES

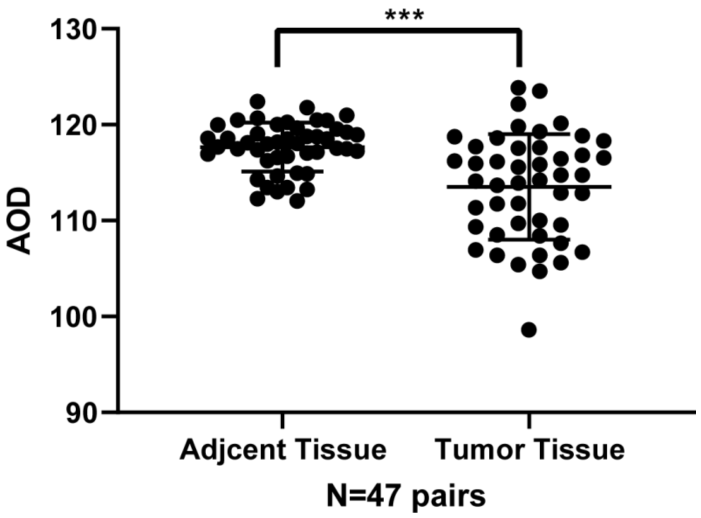

Supplementary Figure 1. Semi-quantitative analysis of the IHC images was conducted by Image-J, and the integral optical density (IOD) and area were collected (N = 47 pairs).

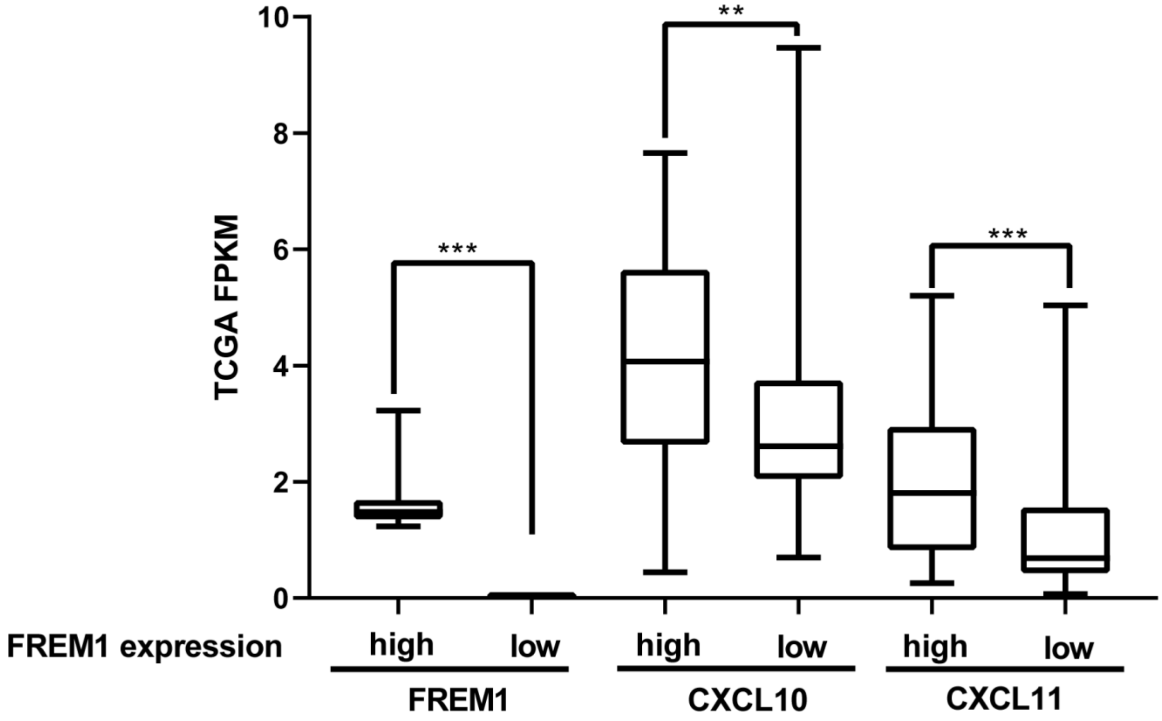

Supplementary Figure 2. The top 50 highest *TILRR*-expressing and top 50 lowest *TILRR*-expressing tumor tissue samples were used (TCGA database) to analyze the expression levels of *CXCL10* and *CXCL11*.

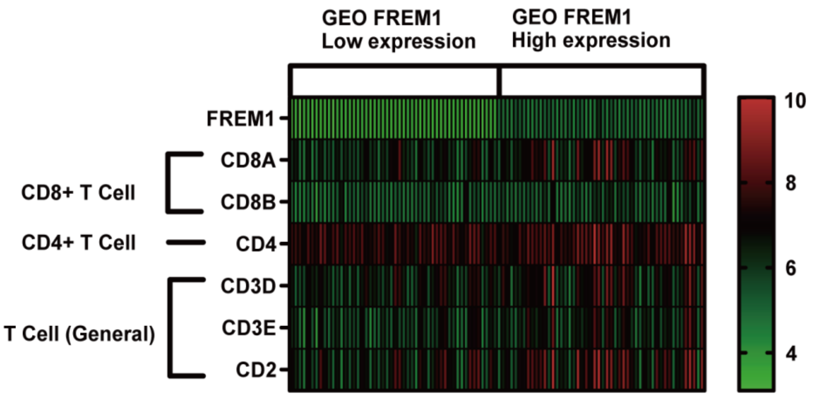

Supplementary Figure 3. The top 50 highest *TILRR*-expressing and top 50 lowest *TILRR*-expressing tumor tissue samples were selected (GEO database) to analyze the expression of marker genes of CD8+, CD4+ and T cells (general).
