# Supplementary material for: TILRR (FREM1 isoform 2) is a prognostic biomarker correlated with immune infiltration in breast cancer
Source: Aging (Albany NY). 2020 Oct 8;12(19):19335–51. doi: 10.18632/aging.103798 (PMC7732299; doi:10.18632/aging.103798)
Supplement: Supplementary Table 1 [file aging-12-103798-s002..docx]

**Supplementary Table 1.** **Correlation analysis between FREM1 and related genes and markers of immune cells in TIMER.**

| **Description** | **Gene markers** | **BRCA** | | | | **Her2** | | | | **BRCA-Basal** | | | | **BRCA-luminal** | | | |
| --- | --- | --- | --- | --- | --- | --- | --- | --- | --- | --- | --- | --- | --- | --- | --- | --- | --- |
|  |  | **None** | | **Purity** | | **None** | | **Purity** | | **None** | | **Purity** | | **None** | | **Purity** | |
|  |  | **Cor** | ***P*** | **Cor** | ***P*** | **Cor** | ***P*** | **Cor** | ***P*** | **Cor** | ***P*** | **Cor** | ***P*** | **Cor** | ***P*** | **Cor** | ***P*** |
| CD8+ T cell | CD8A | 0.498 | *** | 0.495 | *** | 0.729 | *** | 0.697 | *** | 0.346 | *** | 0.378 | *** | 0.542 | *** | 0.545 | *** |
|  | CD8B | 0.431 | *** | 0.430 | *** | 0.703 | *** | 0.684 | *** | 0.253 | * | 0.292 | ** | 0.526 | *** | 0.527 | *** |
| T cell (general) | CD3D | 0.454 | *** | 0.451 | *** | 0.712 | *** | 0.687 | *** | 0.347 | *** | 0.383 | *** | 0.524 | *** | 0.523 | *** |
|  | CD3E | 0.497 | *** | 0.493 | *** | 0.722 | *** | 0.686 | *** | 0.385 | *** | 0.415 | *** | 0.555 | *** | 0.555 | *** |
|  | CD2 | 0.446 | *** | 0.440 | *** | 0.717 | *** | 0.679 | *** | 0.357 | *** | 0.383 | *** | 0.509 | *** | 0.507 | *** |
| B cell | CD19 | 0.420 | *** | 0.411 | *** | 0.685 | *** | 0.682 | *** | 0.434 | *** | 0.456 | *** | 0.473 | *** | 0.460 | *** |
|  | CD79A | 0.479 | *** | 0.469 | *** | 0.707 | *** | 0.700 | *** | 0.408 | *** | 0.428 | *** | 0.559 | *** | 0.547 | *** |
| Monocyte | CD86 | 0.202 | *** | 0.187 | *** | 0.552 | *** | 0.494 | *** | 0.283 | ** | 0.291 | ** | 0.207 | *** | 0.198 | *** |
|  | CD115 (CSF1R) | 0.352 | *** | 0.342 | *** | 0.593 | *** | 0.574 | *** | 0.323 | ** | 0.332 | ** | 0.346 | *** | 0.342 | *** |
| TAM | CCL2 | 0.258 | *** | 0.258 | *** | 0.379 | ** | 0.331 | 0.011 | 0.097 | 0.256 | 0.110 | 0.215 | 0.326 | *** | 0.337 | *** |
|  | CD68 | 0.165 | *** | 0.156 | *** | 0.512 | *** | 0.483 | ** | 0.251 | * | 0.265 | * | 0.165 | *** | 0.160 | ** |
|  | IL10 | 0.235 | *** | 0.218 | *** | 0.563 | *** | 0.509 | *** | 0.274 | * | 0.261 | * | 0.222 | *** | 0.204 | *** |
| M1 Macrophage | INOS (NOS2) | 0.024 | 0.429 | 0.020 | 0.521 | -0.095 | 0.442 | -0.084 | 0.528 | -0.055 | 0.517 | -0.075 | 0.396 | 0.028 | 0.484 | 0.023 | 0.596 |
|  | IRF5 | 0.044 | 0.140 | 0.040 | 0.205 | 0.476 | *** | 0.483 | ** | 0.121 | 0.154 | 0.150 | 0.089 | 0.038 | 0.343 | 0.031 | 0.472 |
|  | COX2 (PTGS2) | 0.465 | *** | 0.465 | *** | 0.266 | 0.030 | 0.232 | 0.077 | 0.083 | 0.329 | 0.041 | 0.643 | 0.597 | *** | 0.604 | *** |
| M2 Macrophage | CD163 | 0.191 | *** | 0.180 | *** | 0.483 | *** | 0.452 | ** | 0.245 | *** | 0.247 | *** | 0.185 | *** | 0.182 | *** |
|  | VSIG4 | 0.167 | *** | 0.165 | *** | 0.256 | 0.036 | 0.235 | 0.073 | 0.122 | 0.042 | 0.146 | 0.019 | 0.162 | *** | 0.166 | ** |
|  | MS4A4A | 0.330 | *** | 0.320 | *** | 0.483 | *** | 0.424 | ** | 0.230 | *** | 0.317 | *** | 0.353 | *** | 0.350 | *** |
| Neutrophils | CD66b (CEACAM8) | 0.027 | 0.370 | 0.036 | 0.252 | -0.030 | 0.809 | -0.090 | 0.499 | 0.100 | 0.239 | 0.134 | 0.131 | 0.055 | 0.173 | 0.055 | 0.203 |
|  | CD11b (ITGAM) | 0.213 | *** | 0.216 | *** | 0.502 | *** | 0.456 | ** | 0.187 | 0.027 | 0.205 | 0.020 | 0.182 | *** | 0.186 | *** |
|  | CCR7 | 0.494 | *** | 0.487 | *** | 0.728 | *** | 0.682 | *** | 0.425 | *** | 0.436 | *** | 0.541 | *** | 0.529 | *** |
| Natural killer cell | KIR2DL1 | 0.188 | *** | 0.183 | *** | 0.378 | * | 0.365 | * | 0.194 | 0.022 | 0.220 | 0.012 | 0.184 | *** | 0.185 | *** |
|  | KIR2DL3 | 0.188 | *** | 0.190 | *** | 0.453 | ** | 0.432 | ** | 0.174 | 0.039 | 0.173 | 0.050 | 0.195 | *** | 0.202 | *** |
|  | KIR2DL4 | 0.152 | *** | 0.142 | *** | 0.409 | ** | 0.375 | * | 0.138 | 0.105 | 0.175 | 0.047 | 0.220 | *** | 0.208 | *** |
|  | KIR3DL1 | 0.267 | *** | 0.263 | *** | 0.517 | *** | 0.457 | ** | 0.280 | ** | 0.276 | * | 0.309 | *** | 0.318 | *** |
|  | KIR3DL2 | 0.280 | *** | 0.279 | *** | 0.478 | *** | 0.453 | ** | 0.246 | * | 0.261 | * | 0.346 | *** | 0.363 | *** |
|  | KIR3DL3 | 0.091 | * | 0.081 | 0.010 | 0.137 | 0.270 | 0.093 | 0.485 | 0.049 | 0.566 | 0.035 | 0.694 | 0.128 | * | 0.137 | * |
|  | KIR2DS4 | 0.200 | *** | 0.202 | *** | 0.241 | 0.049 | 0.186 | 0.159 | 0.139 | 0.101 | 0.156 | 0.078 | 0.248 | *** | 0.265 | *** |
| *(Continued)* | | | | | | | | | | | | | | | | | |
| **Description** | **Gene markers** | **BRCA** | | | | **Her2** | | | | **BRCA-Basal** | | | | **BRCA-luminal** | | | |
|  |  | **None** | | **Purity** | | **None** | | **Purity** | | **None** | | **Purity** | | **None** | | **Purity** | |
|  |  | **Cor** | ***P*** | **Cor** | ***P*** | **Cor** | ***P*** | **Cor** | ***P*** | **Cor** | ***P*** | **Cor** | ***P*** | **Cor** | ***P*** | **Cor** | ***P*** |
| Dendritic cell | HLA-DPB1 | 0.413 | *** | 0.405 | *** | 0.531 | *** | 0.495 | *** | 0.337 | *** | 0.352 | *** | 0.445 | *** | 0.436 | *** |
|  | HLA-DQB1 | 0.271 | *** | 0.272 | *** | 0.463 | *** | 0.429 | ** | 0.227 | ** | 0.220 | 0.012 | 0.298 | *** | 0.302 | *** |
|  | HLA-DRA | 0.364 | *** | 0.359 | *** | 0.553 | *** | 0.515 | *** | 0.310 | ** | 0.326 | ** | 0.381 | *** | 0.377 | *** |
|  | HLA-DPA1 | 0.388 | *** | 0.382 | *** | 0.545 | *** | 0.510 | *** | 0.333 | *** | 0.348 | *** | 0.392 | *** | 0.384 | *** |
|  | BDCA-1 (CD1C) | 0.643 | *** | 0.647 | *** | 0.747 | *** | 0.727 | *** | 0.470 | *** | 0.481 | *** | 0.684 | *** | 0.688 | *** |
|  | BDCA-4 (NRP1) | 0.448 | *** | 0.444 | *** | 0.230 | 0.061 | 0.207 | 0.116 | 0.283 | ** | 0.249 | * | 0.489 | *** | 0.503 | *** |
|  | CD11c (ITGAX) | 0.245 | *** | 0.248 | *** | 0.585 | *** | 0.544 | *** | 0.320 | ** | 0.346 | *** | 0.209 | *** | 0.212 | *** |
| Th1 | T-bet (TBX21) | 0.468 | *** | 0.465 | *** | 0.675 | *** | 0.630 | *** | 0.375 | *** | 0.408 | *** | 0.521 | *** | 0.524 | *** |
|  | STAT4 | 0.508 | *** | 0.497 | *** | 0.714 | *** | 0.682 | *** | 0.404 | *** | 0.422 | *** | 0.542 | *** | 0.533 | *** |
|  | STAT1 | 0.078 | * | 0.070 | 0.027 | 0.406 | ** | 0.379 | * | 0.189 | 0.026 | 0.197 | 0.025 | 0.090 | 0.025 | 0.088 | 0.041 |
|  | IFN-γ (IFNG) | 0.242 | *** | 0.239 | *** | 0.536 | *** | 0.510 | *** | 0.244 | * | 0.274 | * | 0.308 | *** | 0.317 | *** |
|  | TNF-α (TNF) | 0.015 | 0.617 | 0.021 | 0.509 | 0.445 | ** | 0.483 | ** | -0.027 | 0.749 | -0.006 | 0.947 | 0.015 | 0.708 | 0.012 | 0.782 |
| Th2 | GATA3 | -0.122 | *** | -0.116 | ** | -0.058 | 0.641 | -0.052 | 0.692 | -0.033 | 0.698 | -0.000 | 0.996 | -0.299 | *** | -0.300 | *** |
|  | STAT6 | 0.232 | *** | 0.246 | *** | -0.119 | 0.338 | -0.208 | 0.115 | 0.056 | 0.509 | 0.053 | 0.550 | 0.224 | *** | 0.238 | *** |
|  | STAT5A | 0.358 | *** | 0.356 | *** | 0.562 | *** | 0.563 | *** | 0.032 | 0.707 | 0.037 | 0.678 | 0.384 | *** | 0.388 | *** |
|  | IL13 | 0.180 | *** | 0.184 | *** | 0.326 | ** | 0.286 | 0.028 | 0.203 | 0.016 | 0.224 | 0.011 | 0.176 | *** | 0.170 | *** |
| Tfh | BCL6 | 0.192 | *** | 0.206 | *** | 0.001 | 0.992 | -0.002 | 0.989 | 0.201 | 0.017 | 0.203 | 0.021 | 0.125 | * | 0.135 | * |
|  | IL21 | 0.204 | *** | 0.198 | *** | 0.461 | *** | 0.456 | ** | 0.344 | *** | 0.344 | *** | 0.257 | *** | 0.257 | *** |
| Th17 | STAT3 | 0.161 | *** | 0.174 | *** | 0.165 | 0.182 | 0.160 | 0.225 | 0.032 | 0.709 | 0.048 | 0.592 | 0.137 | ** | 0.153 | ** |
|  | IL17A | 0.108 | ** | 0.099 | * | 0.205 | 0.096 | 0.104 | 0.433 | 0.095 | 0.266 | 0.122 | 0.170 | 0.152 | ** | 0.158 | ** |
| Treg | FOXP3 | 0.238 | *** | 0.278 | *** | 0.559 | *** | 0.520 | *** | 0.318 | ** | 0.327 | ** | 0.261 | *** | 0.264 | *** |
|  | CCR8 | 0.189 | *** | 0.180 | *** | 0.479 | *** | 0.423 | ** | 0.358 | *** | 0.353 | *** | 0.194 | *** | 0.194 | *** |
|  | STAT5B | 0.358 | *** | 0.370 | *** | 0.506 | *** | 0.521 | *** | 0.213 | 0.011 | 0.213 | 0.015 | 0.263 | *** | 0.280 | *** |
|  | TGFβ (TGFB1) | 0.344 | *** | 0.346 | *** | 0.304 | 0.013 | 0.345 | * | 0.359 | *** | 0.377 | *** | 0.319 | *** | 0.311 | *** |
| T cell exhaustion | PDCD1 | 0.377 | *** | 0.371 | *** | 0.633 | *** | 0.591 | *** | 0.264 | * | 0.294 | ** | 0.450 | *** | 0.443 | *** |
|  | CTLA4 | 0.285 | *** | 0.281 | *** | 0.616 | *** | 0.560 | *** | 0.345 | *** | 0.364 | *** | 0.305 | *** | 0.304 | *** |
|  | LAG3 | 0.087 | * | 0.076 | 0.017 | 0.425 | ** | 0.359 | * | 0.183 | 0.030 | 0.219 | 0.013 | 0.131 | * | 0.117 | * |
|  | HAVCR2 | 0.176 | *** | 0.169 | *** | 0.501 | *** | 0.461 | ** | 0.289 | ** | 0.302 | ** | 0.163 | *** | 0.163 | ** |
|  | GZMB | 0.285 | *** | 0.277 | *** | 0.555 | *** | 0.523 | *** | 0.183 | 0.031 | 0.212 | 0.159 | 0.395 | *** | 0.396 | *** |

*BRCA, breast invasive carcinoma; TAM, tumour-associated macrophage; Th, T helper cell; Tfh, Follicular helper T cell; Treg, regulatory T cell; Cor, R value of Spearman’s correlation; None, correlation without adjustment. Purity, correlation adjusted by purity.*

**P < 0.01; **P < 0.001; ***P < 0.0001.*
